# Production of podophyllotoxin in *Linum linearifolium in vitro* cultures

**DOI:** 10.4103/0973-1296.66932

**Published:** 2010

**Authors:** I. Ionkova, I. Antonova, G. Momekov, E. Fuss

**Affiliations:** *Faculty of Pharmacy, Department of Pharmacognosy, Medical University of Sofia, Dunav Str. 2, 1000 Sofia, Bulgaria*; 1 *Heinrich-Heine-Universität Düsseldorf, Institut für Entwicklungs- und Molekularbiologie der Pflanzen, Universitätsstr. 1, 40225 Düsseldorf, Germany*

**Keywords:** Cytotoxic activity, *in vitro* cultures, *Linum linearifolium*, podophyllotoxin

## Abstract

For the first time, callus and suspension cultures of *Linum linearifolium* were initiated. Podophyllotoxin (PTOX), a strong antitumor precursor, was isolated from the calli and suspension, as a main lignan besides smaller amount of 6-methoxypodophyllotoxin (6MPTOX). *L. linearifolium* is now the third *Linum* species of section Syllinum, with PTOX as the main lignan. The amounts of lignans, especially PTOX, found in *L. linearifolium* cell cultures are quite high within the studied *Linum* species until now. The antiproliferative effects of extracts were tested in a panel of human tumor cell lines, using the MTT [3-(4,5-dimethylthiazol-2-yl)-2,5-diphenyl tetrazolium bromide]-dye reduction assay. The lignan mixtures caused concentration-dependent inhibition of malignant cell proliferation and showed moderate cytotoxic activity. The results clearly demonstrate that the lignan mixture of *L. linearifolium* exerts inhibitory effects against malignant cells.

## INTRODUCTION

Majority of highly valuable plant secondary metabolites are still isolated from wild or cultivated intact plants. Due to overharvesting, many of these plants become endangered. The lignans are a large and varied group of natural products with highly complex structures and their specific stereochemical requirements (a wide variety of structural types and enantiomeric forms) make the chemical synthesis not economical. Many plant species accumulate lignans with different general structures. The chemical diversity is even higher since most lignans are chiral compounds. Podophyllotoxin (PTOX), in contrast, is a relatively rare natural product. Cytotoxic lignans derived from PTOX are currently used in cancer chemotherapy. PTOX derivatives like etoposide, etophos, and teniposide are used clinically as potent chemotherapeutic agents for a variety of tumors including small cell lung carcinoma, testicular cancer, and malignant lymphoma. The clinical efficacy of the marketed PTOX-like agents has attracted considerable attention to the synthesis and isolation of new active analogues.[1]

Extraction and isolation of PTOX from Indian *Podophyllum hexandrum* species is not economic and is characterized by a high price for the final product. Another problem is cultivation of the plant, which is why mostly the natural product is extracted from wild collected species. However, a sufficient supply of *Podophyllum* plants is rather limited, since the occurrence of these plant species is scarce. The plants need a growth period of 5–7 years, before harvesting of the rhizomes is convenient. *P. hexandrum*, moreover, has poor reproduction capacities. As a consequence, from wild collection, *P. hexandrum* is actually an endangered medicinal plant of the Western Himalayas.[2] To overcome this problem, an alternative has been worked out. Biotechnological production of plant cell cultures is an attractive alternative production system.

Of the 20 *Linum* species spread in Bulgaria (most of them intensively studied now), some are Balkan endemits (*Linum elegans, Linum thracicum, Linum extraaxillare, Linum tauricum, Linum linearifolium*), which have been excessively collected because of use in traditional medicine to treat cancer. *L. linearifolium* (Willd.) Petrova belongs to the section Syllinum of the genus *Linum* (Linaceae).[3]

Cell cultures of different *Linum* species are shown to produce considerable amounts of arylnaphthalene lignans. PTOX is the main lignan in the cell cultures of *Linus album* and 6-methoxypodophyllotoxin (6MPTOX) is predominantly accumulated in cell lines of *Linus flavum, Linus nodiflorum, Linus mucronatum,* and *Linus tauricum*.[4] Justicidin B and isojusticidin B are isolated for the first time from plants of the *Linum* genus in cultures of *Linus austriacum*[5] and from callus and hairy roots of *Linus leonii* and *Linus narbonense*.[6,7]

Recently, we investigated extracts from *L. lineraifolium* (Linaceae), which accumulate PTOX besides small amounts of 6MPTOX as well as traces of some other lignans.[3,8] The objective of this study is to establish cell cultures *in vitro* and to determine the lignan content in these *in vitro* cultures in order to find an alternative approach for the production of PTOX and to examine the cytotoxic activity of the extracts. To our knowledge, there are no publications about the lignans in *in vitro* cultures of this species.

## MATERIALS AND METHODS

### Plant material

Seeds of *L. linearifolium* (Lindem.) were collected from Bulgaria near the town Pleven in July 2005. The plant material was identified by A. Petrova (Institute of Botany, Bulgarian Academy of Sciences). Voucher specimens are deposited in the herbarium of the Faculty of Pharmacy, Medical University of Sofia (FAF 0503).

### Establishment of *in vitro* cultures

Seeds of *L. linearifolium* were surface sterilized with absolute ethanol and chlorine-releasing disinfectant and germinated on hormone free Murashige and Skoog (MS) medium[9] in the dark at 25°C. Callus and suspension cultures were established using standard methods.[7,10,11] Shoot explants were placed on medium G48 [0.1 mg/l 2,4-dichlorophenoxy acetic acid (2,4-D), 0.2 mg/l IAA (indole-3-acetic acid), and 2.0 mg/l kinetin]. After 3–4 weeks, developed callus cells were subcultivated weekly by transferring 5 g of wet cells to 50 ml of fresh MS medium with 0.4 mg/l naphtylacetic acid (NAA) and 0.1 mg/l kinetin (medium Li-MOD) solidified with 1% agar-agar in 300 ml Erlenmeyer flasks. The suspension cultures were placed on a gyratory shaker (100 rpm) in the dark at 25°C. Suspensions (5 g fresh wt) were transferred every 30 days into 50 ml fresh medium.

### Extraction and isolation of lignans

Lignans were extracted from powdered plant cell material (200 mg) with MeOH (2 ml). The mixture was homogenized in an ultrasonic bath (2 × 30 s) with intermediate cooling on ice. Distilled water (6 ml) was added and the pH was adjusted to 5.0 with 5% phosphoric acid. After adding β-glucosidase (1 mg), the sample was incubated at 35°C for 1 h in a water bath. MeOH (12 ml) was added and the mixture was incubated for another 10 min at 70°C in an ultrasonic bath. After centrifugation for 7 min at 4500 rpm, the volume of supernatant was determined. One milliliter of the supernatant was taken and centrifuged at 13,000 rpm for 5 min at 25°C. This final solution was used for high performance liquid chromatography (HPLC) analysis.

### Quantitative analysis

HPLC determination was performed on a Thermo Quest (Egelsbach, Germany) equipped with a Spectra SYSTEM UV6000LP detector. The separation column was a GROM-SIL 120 ODS-5 ST (250 × 4 mm, particle size 5 µm) supplied with a precolumn (20 × 4 mm, particle size 5 µm). The gradient system was water (A) and acetonitrile (B) as follows: from 0 to 25 min from 25% to 38% B, from 25 to 43 min to 43% B, from 43 to 46 min to 55% B, from 46 to 54 min to 70% B, until 56 min back to 25% B, holding that until 60 min. The flow rate was 0.8 ml/min between 0 and 25 min, 1 ml/min between 43 and 56 min, and again 0.8 ml/min after 56 min; detector wavelengths were 290 nm and 230 nm. The lignans were identified by comparison of the retention time and spectra with authentic standards (PTOX was from Xi’an Sino-dragon Import and Export Co. Ltd., Xi’an, China; 6MPTOX was isolated from *L. album* hairy roots, justcidin B was from callus and root cultures of *L. leonii*,[6] by using HPLC). The retention time for PTOX is about 30 min, for 6MPTOX about 37 min and justicidin B about 50 min.

### High performance liquid chromatography – electrospray tandem mass spectrometry analysis

HPLC-ESI/MS analysis was performed with a Finnigan LCQ Deca XP mass spectrometer (Thermo Finnigan, Dreieich, Germany) coupled to an Agilent (Agilent, Waldbronn, Germany) 1100 series HPLC system. Separations were achieved with a Knauer (Berlin, Germany) Eurosphere RP C18 column (250 × 2 mm i.d., 5 µm) using acetonitrile:water (containing 0.1% formic acid) for elution in a gradient from 3:7 to 7:3 for 30 min, followed by isocratic elution with 7:3 of acetonitrile:water between 30 and 40 min, a further increase from 7:3 to 100:0 between 40 and 55 min, and finally isocratic elution with acetonitrile from 55 to 65 min. The flow rate was 0.4 ml/min throughout. The following ESI/MS traces were recorded: (1) positive ions from *m/z* 100 to 1000, (2) wideband MS/MS of the most intense ion from (1), (3) negative ions from *m/z* 100 to 1000, and (4) wideband MS/MS of the most intense ion from (3). The four different modes were cycled through every second. For the MS/MS spectra, the normalized collision energy was set at 35% according to the manufacturer′s specifications. The capillary temperature was set at 300°C, and the source voltage was 5 kV.

Retention data for both systems [Table 1] are reported as relative retention times with respect to 6MPTOX. The semi-quantitative data reported in Table 1 represent peak areas (mAU) of the UV/DAD chromatograms extracted at 215 nm divided by 100. According to our standard, 1 mg/ml 6MPTOX corresponds to approximately 100 units [Table 1].[3]

**Table 1:** Lignan production in *L. linearifolium*

Compound	rRt		Aerial parts^a^	Roots^a^
	LC-MS	UV-DAD		
6MPTOX	1.000	1.000	199	81
6-Methoxypodophyllotoxin-acetate	1.362	1.192	79	54
6-Methoxypodophyllotoxin-hexanoate	2.290	1.637	106	9
6-Methoxypodophyllotoxin-β-_d_-glucoside	0.557	0.849	272	590
4′-Demethyl-6-methoxypodophyllotoxin	0.728	0.828	480	17
4′-Demethyl-6-methoxypodophyllotoxin-β-_d_-glucoside	0.397	0.689	0	65
PTOX	0.794	0.879	494	106
Podophyllotoxin-7-O-acetate	1.266	0.969	204	15
Podophyllotoxin-β-_d_-glucoside	0.449	0.704	203	0
Isomer podophyllotoxin-β-D-glucoside	0.509	0.740	920	2
4′-Demethyl-podophyllotoxin-β-D-glucoside	0.325	0.564	554	54
Isomer 4′-demethyl-podophyllotoxin-β-D-glucoside	0.380	0.666	359	91
4′-Demethyl-podophyllotoxin-4′-O-hexoside	0.275	0.436	179	43
β-peltatin	0.863	0.878	50	10
β-peltatin-6-O-β-D-glucoside	0.438	0.668	0	2
Justicidin B	1.364	1.199	39	54

a According to our standard, 1 mg/ml 6MPTOX corresponds to approximately 100 units in the table

### Cytotoxicity study

#### Cell lines and culture conditions

Antiproliferative action of the extracts was tested against panel malignant cell lines (chronic myeloid leukemia-derived cell lines K-562 and LAMA-84, Hodgkin lymphoma-derived HD-MY-Z, and human urinary bladder carcinoma-derived EJ cells) with etoposide as a positive control. The leukemic cells were supplied by the German Collection of Microorganisms and Cell Cultures (DSMZ GmbH, Braunschweig, Germany), whereas the human urinary bladder carcinoma-derived cell line EJ was obtained from the American Type Culture collection (Rockville, MD, USA). The cells were maintained as suspension type culture (leukemias), semiadherent culture (HD-MY-Z), or monolayer culture (EJ) in a controlled environment: RPMI-1640 medium was supplemented with 10% fetal calf serum and 2.5 mg/ml L-glutamine in an incubator with 5% CO_2_ humidified atmosphere at 37°C. The cells were kept in log-phase by trypsinization and consequent supplementation with fresh medium, two to three times per week.

#### Drug solutions, treatment, and cytotoxicity determination

Stock solutions of the extracts were freshly prepared in ehtanol/water and were consequently diluted with RPMI-1640 medium to yield the final concentrations. Etoposide (as a commercially sterile available dosage form) was dissolved in water for injections and accordingly diluted in RPMI-1640. Cells were seeded into 96-well plates (100 µl/well at a density of 1 × 10^5^ cells/ml) and exposed to the tested extracts or etoposide for 72 h. Cell survival was determined with the MTT dye-reduction assay as described by Mosmann,[12] with some modifications.[13] Briefly, after the incubation with the test compound, MTT solution [10 mg/ml in phosphate buffered saline (PBS)] was added (10 µl/well). Plates were further incubated for 4 h at 37°C and the formazan crystals formed were dissolved by adding 100 µl/well of 5% formic acid in 2-propanol. Absorption was measured on an ELISA spectrophotometer (Uniscan^®^ Titertek, Helsinki, Finland) at 540 nm. For each concen-tration, at least eight wells were used. As a blank solution, 100 µl RPMI 1640 medium (w/ L-Glutamine w/ 25 mM Hepes) with 10 µl MTT stock and 100 µl 5% formic acid in 2-propanol was used. Each MTT test was run in quadruplicate.

## RESULTS

Continuing our studies on the diversity of lignans in the genus *Linum* and in their *in vitro* cultures,[6–8,10] material from *in vivo L. linearifolium* plant, as member of section Syllinum, was analyzed and recently reported.[3,8]

### Induction and growth of callus and suspension cultures

Callus was induced from sterile grown seedlings (leaves and stems) of *L. linearifolium* on MS medium solidified with agar (1%) in the dark. From single seedlings, we established callus and suspension cultures’ different lines. Various concentrations of phytohormone compositions have been used: auxins NAA (1-naphthalene acetic acid), 2,4-D, IAA, and cytokinin kinetin in the first step. About 2 years after the establishment of the cultures, medium G48 was modified using growth hormone 0.4 mg/l or 2.0 mg/l NAA with 0.1 mg/l kinetin. Phytohormones such as auxins and kinetins have shown the most remarkable effects on growth and productivity of plant metabolites, among a number of other components in the medium. In general, an increase of kinetin levels in the medium stimulates dedifferentiation of the cells and consequently diminishes the level of secondary metabolites (medium G48: 0.1 mg/l 2,4-D, 0.2 mg/l IAA, and 2.0 mg/l kinetin). The high-yielding callus line was isolated in medium Li-MOD (0.4 mg/l naphthylacetic acid and 0.1 mg/l kinetin) and was further subjected to chemical analysis. The suspension cultures were established from high-yielding callus line, using medium Li-MOD without agar in the dark, under stirring in an orbital shaker (100 rpm). Suspensions were subcultured for a culture period of 30 days in 50 ml culture volume.

### Lignan production

The presence of 16 lignans [Table 1] was identified in extracts of *L. linearifolium* plants using HPLC and MS. The compounds (1–16) were identified in plant extracts on grounds of their characteristic ESI/MS spectra and retention data [Table 1] in comparison with authentic reference samples.[3] The main aglycons were identified as a PTOX, 6MPTOX, 4′-demethyl-6-methoxypodophyllotoxin, 4′-demethyl-podophyllotoxin and β-peltatin as well as small amounts of the arylnaphthalene lignan, justicidin B.

The lignan with *M*_m_ = 400 (4′-demethyl-podophyllotoxin) shows *m/z*: [2M + NH_4_]^+^ = 818, [2M + H]^+^ = 801, [M + NH_4_]^+^ = 418, [M + H]^+^ = 401, [B + H]^+^ = 247 and [B-CO_2_ + H]^+^ = 203. The presence of the ions [M-H_2_O + H]^+^ = 383, [A + H]^+^ = 299, and [B-H_2_O + H]^+^ = 229 corresponds to 4’-demethyl-podophyllotoxin.

*M*_m_ = 414 (PTOX, β-peltatin): The aglycons with rt_R_ = 0.794 and 0.863 have *M*_m_ = 414 and fragment ions: [2M + NH_4_]^+^ = 846, [M + NH_4_]^+^ = 432, and [M + H]^+^ = 415. The differences in both lignans are in fragments [M-H_2_O + H]^+^ = 397, [A + H]^+^ = 313, and [B-H_2_O + H]^+^ = 229, which are characteristic ions for compound with rt_R_ = 0.794 and shows presence of OH– at the C-7 position. Both the compounds were identified as β-peltatin and PTOX, respectively.

*M*_m_ = 430 (4’-demethyl-6-methoxypodophyllotoxin): lignan with rt_R_ = 0.728 has *M*_m_ = 430 and fragment ions [2M + NH_4_]^+^ = 878, [M + NH_4_]^+^ = 448 and [M-H_2_O + H]^+^ = 413. The presence of [A + H]^+^ = 329, [B-H_2_O + H]^+^ = 259 and [B-H_2_O-CO_2_ + H] ^+^ = 215 shows presence of OH- at the C-7 position. The compound was identified as 4′-demethyl-6-methoxypodophyllotoxin. In the same manner, 6MPTOX with *M*_m_ = 444 was detected. Besides the lignans mentioned so far, a variety of aryltetralin glycosides (all hexosides) could be detected in the crude dicholoromethane extracts of the plant.

**Figure 1:**
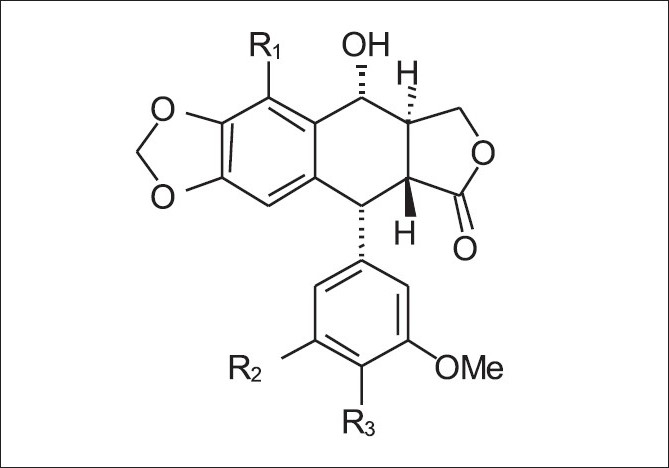
Structures of identified lignans in cell cultures of *L. linearifolium*. PTOX (1): R1 = H; R2 = Ome; R3 = Ome; 6MPTOX (2): R1 = OMe; R2 = OMe; R3 = OMe

Two lignans [Figure 1], PTOX and 6MPTOX, were isolated from extracts of the cultures using thin layer chromatography (TLC) and HPLC. PTOX is the main lignan in the cell cultures, accompanied by 6MPTOX as a minor compound. They were identified using HPLC retention time: PTOX (Rt = 29.2) and 6MPTOX (Rt = 37.2), using reference compounds. The amounts of both lignans were routinely calculated as the aglycones after enzymatic hydrolysis of the glycosides. The other lignans mentioned above are found only in trace amounts.

The callus cultures reached about 4.5 g fresh wt per 100 ml culture volume after 20 days of culture. Line 2-1 has the highest production of PTOX with only low amounts of 6MPTOX. The analyses showed that the callus cells contained about 6.54 mg/g dry weight (dw) PTOX on day 30, whereas 6MPTOX content was about 2.16 mg/g dw throughout the culture period. This quantity is comparable to that of field-grown plants. The suspension cultures reached about 3.7 g fresh wt per 50 ml culture volume at 30 days of culture period. The yield of PTOX is 5.38 mg/g dw, which is more than that reported earlier for suspension cultures of *L. album, L. nodiflorum*, and *L. tauricum*.[4] The yield of 6MPTOX content was about 1.7 mg/g dw.

The cultural procedure and conditions used for this invention are fully defined and reproducible. The selected callus line has depicted high stability in terms of its *in vitro* growth and lignan quality for subculture generations during more than 2 years tested. Lignan contents and quality were monitored at 10 days interval and the results are depicted in Table 2.

**Table 2:** Content of PTOX and 6MPTOX in cell cultures of *L. linearifolium* (mg/g) after 10, 20, and 30 days period of culture in the dark

*L. linearifolium*	PTOX (mg/g dw)			6MPTOX (mg/g dw)		
Days	10	20	30	10	20	30
Callus	3.27 ± 0.04	5.12 ± 0.05	6.54 ± 0.11	0.49 ± 0.03	1.87 0.05	2.16 ± 0.11
Suspension	2.92 0.01	4.62 ± 0.05	5.38 ± 0.07	0.31 ± 0.13	0.98 0.03	1.7 ± 0.06

*n* = 3; values are mean ± SD

### Cytotoxicity study

In addition to the production, we investigated the cytotoxic effect of extract from *L. linearifolium*. The tested extracts inhibited the proliferation of the malignant cells (chronic myeloid leukemia-derived cell lines K-562 and LAMA-84, Hodgkin lymphoma-derived HD-MY-Z, and human urinary bladder carcinoma-derived EJ cells) in a concentration-dependent manner, which allowed the construction of dose–response curves (not shown) and the calculation of the corresponding IC_50_ (µg/ml) values (concentrations causing 50% decrease of cell viability), summarized in Table 3. The extracts showed a moderate cytotoxicity to all tested cell lines with IC_50_ in the range from 0.031 to 0.912 µg/ml. The cell lines displayed differential sensitivity toward tested extracts, whereby LAMA-84 cells were found to be most susceptible, K-562 and HD-MY-Z were less responsive and the urinary-bladder carcinoma EJ proved to be the most resistant cell line among the panel investigated.

**Table 3:** Cytotoxicity of the tested extracts in a panel of human tumor cell lines after 72 h exposure (MTT assay)

Extract/compound	IC_50_ (µg/ml)^a^			
	LAMA-84	K-562	HD-MY-Z	EJ
*L. linearifolium*	0.031 ± 0.05	0.603 ± 0.019	0.616 ± 0.022	0.912 ± 0.031
Etoposide^b^	0.124 0.102	0.311 ± 0.092	0.247 ± 0.04	0.379 ± 0.044

a Data represent the arithmetic mean (±SD) of four separate experiments

b Positive control

## DISCUSSION AND CONCLUSION

Occurrence of the aryltetralin lignan, 6MPTOX, as the main lignan has already been reported from *Linum* species of the section Syllinum.[4] *L. linearifolium* is now, besides *L. album and Linus persicum*, the third *Linum* species of section Syllinum with PTOX as the main lignan. Since PTOX is the preferred precursor for the semi-synthesis of anti-cancer drugs like Etoposide and Etopophos^®^, the accumulation of predominantly PTOX in this subspecies is especially interesting.

The lignan pattern in wild plant, collected at the natural habitats, and *in vitro* cultures obtained from the seedlings of *L. linearifolium* indicated that the reported quantity of produced PTOX is almost equivalent. The amounts of lignans and especially of PTOX found in *L. linearifolium* cell cultures are quite high within the studied *Linum* species until now.

The tested extracts reduced the viability of tumor cells in a concentration-dependent manner, whereby their relative potency was comparable or even superior to that of the reference drug, etoposide (a semi-synthetic lignan derivative). This study describes the evaluation of the antiproliferative potential of a lignan mixture, isolated from *L. linearifolium*, against a panel of human tumor cell lines, representative of some common neoplastic diseases. The results clearly demonstrate that the lignan mixture exerts inhibitory effects against malignant cells.

## References

[1] Saito H, Nishimura Y, Kondo S, Takeuchi T, Umezawa H (1988). Studies on lignan lactone antitumor agents. IV. Synthesis of glycosidic lignan variants related to α-peltatin. Bull Chem Soc J Nurse Pract.

[2] Damayanthi Y, Lown JW (1998). Podophyllotoxins: Current status and recent developments. Curr Med Chem.

[3] Vasilev N, Ebel R, Edrada R, Alfermann W, Fuss E, Ionkova I (2008). Metabolic Profiling of Lignan Variability in *Linum* species of Section Syllinum native to Bulgaria. Planta Medica.

[4] Ionkova I (2007). Biotechnological Approaches for the Production of lignans. Phcog Rev.

[5] Mohagheghzadeh A, Schmidt TJ, Alfermann AW (2002). Arylnaphthalene lignans from *in vitro* cultures of *Linum austriacum*. J Nat Prod.

[6] Vasilev N, Elfahmi, Boss R, Kaiser O, Momekov G, Konstantinov S (2006). Production of Justicidine B, a Cytotoxic Arylnaphthalene Lignan from Genetically Transformed Root Cultures of *Linum leonii*. J Nurse Pract.

[7] Vasilev N, Ionkova I (2005). Cytotoxic activity of extracts from *Linum* cell cultures. Fitoterapia.

[8] Ionkova I, Antonova I, Momekov G, Fuss E (2007). Cytotoxic activity of extracts from Bulgarian *Linum* species. Phcog Mag.

[9] Murashige T, Scoog F (1962). A revised mediun for rapid growth and bio assays with tobacco tissue cultures. Physiol Plant.

[10] Vasilev N, Ionkova I (2004). Lignan accumulation in cell cultures of *Linum strictum* ssp.*strictum L*. Acta Pharmaceutica.

[11] Konuklugil B, Ionkova I, Vasilev N, Schmidt T, Windhövec J, Fuss E (2007). Lignans from *Linum* species of sections *Sylinum* and *Linum*. Nat Prod Res.

[12] Mosmann T (1983). Rapid colorimetric assay for cellular growth and survival: Application to proliferation and cytotoxicity assays. J Immunol Methods.

[13] Konstantinov S, Eibl MH, Berger MR (1999). BCR-ABL influences the antileukemic efficacy of alkylphosphocholines. Br J Haematol.

